# Effects of condensates from volcanic fumaroles and cigarette smoke extracts on airway epithelial cells

**DOI:** 10.1007/s13577-023-00927-1

**Published:** 2023-06-12

**Authors:** Caterina Di Sano, Serena Di Vincenzo, Doriana Lo Piparo, Claudia D’Anna, Simona Taverna, Valentina Lazzara, Paola Pinto, Francesco Sortino, Elisabetta Pace

**Affiliations:** 1 Istituto di Farmacologia Traslazionale (IFT), Consiglio Nazionale delle Ricerche, Via Ugo La Malfa, 153, 90146 Palermo, Italy; 2 Istituto per la Ricerca e l’Innovazione Biomedica (IRIB), Consiglio Nazionale delle Ricerche, Palermo, Italy; 3 Dipartimento Promozione della Salute, Materno-Infantile, di Medicina Interna e Specialistica di Eccellenza “G. D’Alessandro” (PROMISE), Università degli Studi di Palermo, Palermo, Italy; 4 Istituto Nazionale di Geofisica e Vulcanologia (INGV), Palermo, Italy

**Keywords:** Volcanic fumaroles, Airway epithelial cells, Homeostasis, Injury, Inflammation, Cigarette smoke

## Abstract

The impact of volcanic airborne products on airway epithelium homeostasis is largely unknown. This study assessed the effects of volcanic Fumarole Condensates (FC) alone or combined with Cigarette Smoke Extracts (CSE) on airway epithelial cells (16HBE and A549). Chemical composition of FC was analyzed by gas chromatography and HPLC. Cells were exposed to FC and IL-33 and IL-8 were assessed. The effects of FC and CSE on cell injury were evaluated assessing cell metabolism/cell viability, mitochondrial stress, cell apoptosis/cell necrosis, and cell proliferation. FC contained: water vapor (70–97%), CO_2_ (3–30%), acid gases (H_2_S, SO_2_, HCl, HF) around 1%. FC increased the intracellular IL-33 but differently modulated IL-33 and IL-8 gene expression and IL-8 release in the tested cell lines. FC without/with CSE: (a) increased cell metabolism/cell viability in 16HBE, while decreased it in A549; (b) increased mitochondrial stress in both cell types. FC with CSE increased cell necrosis in A549 in comparison to CSE alone. CSE reduced cell proliferation in 16HB,E while increased it in A549 and FC counteracted these effects in both cell types. Overall, FC induce a pro-inflammatory profile associated to a metabolic reprogramming without a relevant toxicity also in presence of CSE in airway epithelial cells.

**Supplementary Information:**

The online version contains supplementary material available at 10.1007/s13577-023-00927-1.

## Introduction

A relevant percentage (10%) of the worldwide population lives in the proximity of an active volcano. However, volcanogenic air pollution studies are still limited when compared with studies evaluating air pollution due to anthropic impact. Therefore, the volcanogenic air pollution up to date represents an unknown risk to human populations living in volcanic areas worldwide.

Vulcano is a volcanic island that belongs to the Aeolian archipelago in Sicily (Italy). Vulcano is an inhabited island with resident citizen and in summer is a destination for many tourists. Its active cone (La Fossa) erupted for the last time in 1888–1890 [1]. After this eruption, the volcano emitted, from the La Fossa crater, only fumaroles composed of water and acid gases (SO_2_, H_2_S, HCl, HF), traces of gaseous elements (He, Ne) and metallic trace elements [2–4].

The relationship between geology and human health is a recent research area. The beneficial or detrimental impact of Vulcano fumaroles on airway epithelium is unknown. The airway epithelium represents a mechanical, chemical and immunological barrier against environmental insults such as particles, pollutants and microbes [5]. Cigarette smoke is the main risk factor for many airway diseases such as Chronic Obstructive Pulmonary Disease (COPD) and lung cancer [6]. Cigarette smoke increases IL-8 release in 16HBE and in A549 cells and reduces IL-33 release and promotes the intracellular cytoplasmic accumulation of the full length forms of IL-33 in bronchial epithelial cells [7]. Airway epithelium of proximal and distal airways shows differences in responses to injury [8–10]. In this regard, it has been established that Cigarette Smoke Extract (CSE) increases STAT-3 nuclear expression and Ki-67 proliferation marker in bronchial epithelial cells (16HBE) but not in alveolar epithelial cells (A549) [9].

The present study aimed to assess the effects of the “condensates” of the fumaroles (FC) in human bronchial epithelial cell line (16HBE) and in alveolar cell line (A549). We used the 16HBE cell line to create an in vitro model of proximal airway epithelium and, although the A549 cell line arises from a human lung adenocarcinoma, it represents a model of distal airway epithelium, useful to investigate functions and actions of alveolar cell in vitro [11, 12]. The fumaroles named F5 HT (high temperature) characterized for the elevated temperature of the released gases and collected as previously described [13] at different times from the La Fossa crater of the Vulcano Island, were used. The fumarole gases were collected with the method proposed by Giggenbach et al. [14] and Sortino et al. [13] and at the same time, the “condensates” that were used for the experiments were generated. Chromatographic techniques (GC and HPLC) and titration techniques described by Sortino et al. [13] were used for the analysis of the samples.

The direct effect of FC on IL-33 gene expression, intracellular protein expression and release and IL-8 gene expression and protein release was explored. In some experiments, the effects of co-exposure with FC and CSE were investigated by assessing cell viability/metabolism, cell apoptosis/necrosis, mitochondrial stress and clonogenic activity.

## Materials and methods

### Collection and composition of volcanic fumaroles

Volcanic fumarole condensates were collected at different times from the F5 fumarole (F5HT; HT = high temperature) in Vulcano Island (Aeolian Islands, southern Italy). The sampling of fumarolic gases was carried out by the Giggenbach et al. 1975 method [14], which can be easily applied to fumaroles where air contamination is very low. The purpose of sampling was to take the gases directly from the fumarole, preventing them from being contaminated by the air; to do this, a series of measures were implemented. Detailed method to obtain condensate of fumarole was reported in Supplementary Materials (S1).

The composition of FC was evaluated by different analytical techniques (Gas chromatography and HPLC titration) as previously reported [13, 15].

### Preparation of CSE

The smoke produced by the combustion of two cigarettes, from which the filter was removed, (Kentucky 3R4F—The Tobacco Research Institute, University of Kentucky) was drawn in 20 ml of PBS (Phosphate-Buffered Saline) using a peristaltic pump Watson-Marlow 323 E/D (Rotterdam, The Netherlands), set at 70 rpm as previously described [16]. The CSE solution was filtered using a 0.22 μm pore filter to remove bacteria and large molecules. This method produces a more uniform puff profile and allows to standardize the CSE solution in order to improve reproducibility. The solution was considered to be 100% of CSE and diluted at desired concentration in each experiment. The concentration of CSE was measured using spectrophotometer as previously described [16] at the 320 nm wavelength. The difference of absorbance, between the diverse experiments, was low and the mean OD of the diverse preparation was 1.32 ± 0.15 [17].

### Cell cultures

16HBE, an immortalized normal bronchial epithelial cell line that retains the differentiated morphology and function of normal airway epithelial cells, and A549 (Interlab Cell Line Collection, Genoa, Italy), bronchioloalveolar cell line derived from a patient with a lung adenocarcinoma, were used in this study. 16HBE and A549 cells were maintained in a humidified atmosphere of 5% CO_2_ in air at 37 °C and were cultured as adherent monolayers. Eagle’s Minimum Essential Medium (MEM), supplemented with 10% heat-inactivated (56 °C, 30 min) fetal bovine serum (FBS), 1% non-essential aminoacids, 2 mM L-glutamine and 0.5% gentamicin (all from Euroclone, Pero (MI), Italy) was used for 16HBE. RPMI medium supplemented with heat-inactivated (56 °C, 30 min) 10% FBS, penicillin–streptomycin, 1% non-essential aminoacids and 2 mM L-glutamine (all from Euroclone, Pero (MI), Italy) was used for culturing A549.

### Cell stimulation

16HBE and A549 cells were cultured to confluence, then the serum was reduced from 10 to 1% in the medium and the cells were stimulated with different concentration of FC (2, 5 and 10%) and with CSE (10% for 16HBE and 2.5% for A549) [9, 17, 18]. At the end of stimulation, cells were collected for further evaluations. At least three replicates were performed for each experiment. The pH measured by pH Test Strip (P-4536 Lot. 010B164536, Sigma-Aldrich, St Louis, MO) did not vary among the different experimental conditions (pH 7.5).

### Intracellular IL-33 expression

The expression of IL-33 in 16HBE and in A549 was evaluated by flow-cytometry using a FACS Calibur (Becton Dickinson, Mountain View, CA). To evaluate the expression of intracellular IL-33 before incubation with mouse monoclonal antibody, cells were treated overnight with GolgiStop (2 μM final concentration) (BD PharMingen) and fixed with PBS containing 4% paraformaldehyde for 20 min at room temperature. Fixed cells were washed twice in permeabilization buffer (PBS containing 1% FBS, 0.3% saponin, and 0.1% Na azide) for 5 min at 4 °C, then incubated with a mouse monoclonal IgG antibody anti-IL-33 (Adipogen) (1:100; for 1 h) followed by a FITC conjugated anti-mouse IgG (Dako, Glostrup, Denmark). Negative controls were performed using mouse immunoglobulins negative control (Dako). Data are expressed as percentage of positive cells.

### Real-time PCR

Total cellular RNA was extracted from 16HBE and A549 using TRIzol Reagent (Invitrogen, Carlsbad, CA, USA) and reverse-transcribed to cDNA, using iScript cDNA Synthesis kit (Biorad, Hercules, CA, USA). Real-time quantitative PCR of IL-8 and IL-33 gene was carried out on Step One Plus Real-time PCR System (Applied Biosystems, Foster City, CA, USA) using specific FAM-labeled probe and primers (prevalidated TaqMan Gene expression assay for IL8, Hs00174103m1 and prevalidated TaqMan Gene expression assay for IL-33, Hs01125943m1; Assays on Demand, Applied Biosystems) as previously described [10, 19]. IL-8 and IL-33, gene expression was normalized to GAPDH endogenous control gene. Relative quantification of mRNA was carried out with comparative CT method (2^−ΔΔCT^) and was plotted as fold-change compared to Non-treated (NT) sample, that were chosen as the reference sample.

### IL-8 and IL-33 release by ELISA

The concentrations of IL-8 and of IL-33 were determined with Enzyme-Linked Immunosorbent Assays (ELISA) (Duoset; R&D Systems, Minneapolis, MN).

### Cell viability/metabolic assay

Cell viability was evaluated by CellTiter 96 Aqueous One Solution Cell Proliferation Assay, (PROMEGA, Madison WI USA) as previously reported [20] and according to the manufacturer’s instructions. One Solution reagent contains MTS [3-(4,5-dimethylthiazol-2-yl)-5-(3-carboxymethox-yphenyl)-2-(4-sulfopheyl)2H-tetrazolium]. Cells were plated in 96-well plates and were treated for 24 h with FC with/without CSE; 20 μL of One Solution reagent was added to each well, and incubated for 20 min at 37 °C, 5% CO_2_. The absorbance was read at 490 nm on the Microplate reader wallacVictor2 1420 Multilabel Counter (PerkinElmer, Milan, Italy). Results are expressed as percentage relative to no treated (NT) sample.

### Measure of mitochondrial superoxide

The production of mitochondrial superoxide was evaluated using MitoSOX™ Red mitochondrial superoxide indicator (Molecular Probes Waltham, MA, USA) [19]. 16HBE cells were seeded in a 12-well plate and incubated with FC (10%) and/or with CSE (10%) for 3 h. A549 were seeded in 12-well plate and stimulated with FC (10%) and/or with CSE (2.5%) for 3 h. Then, the cells were harvested, washed with PBS and stained with 3 μM MitoSOX Red probe for 15 min at 37 °C. At the end of the incubation, the cells were washed with PBS and analyzed by flow-cytometry using CytoFLEX (BeckmanCoulter, Brea, CA, USA).

### Cell apoptosis by annexin V-binding method

16HBE cells were seeded in a 12-well plate and incubated with FC (10%) and/or with CSE (10%) for 24 h. A549 were seeded in 12-well plate and stimulated with FC (10%) and/or with CSE (2.5%) for 24 h. Cell apoptosis was evaluated by staining with annexin V-fluorescein isothiocyanate and propidiumiodide (PI) using a commercial kit (Bender Med- System, Vienna, Austria) following the manufacturer’s directions. Cells were analyzed using a FACSCalibur (Becton Dickinson, Mountain View, CA) analyzer equipped with an Argon ion Laser (Innova 70 Coherent) and Consort 32 computer support.

PI-negative and annexin V-negative cells, allocated in the lower left quadrant, were identified as viable cells; PI-positive cells, present in the upper left quadrant, were identified as necrotic cells; the late apoptotic cells were identified as PI and annexin V double-positive cells in the upper right quadrant and the single annexin V-positive cells, present in the lower right quadrant, were identified as early apoptotic cells.

### Clonogenic assay

Clonogenic assay was performed as previously described [21] to evaluate the cell proliferation. The cell lines were stimulated in 1% FBS medium with the different treatments, as previous described, for 24 h; then, they were harvested and seeded in a 12-well plate at a clonogenic density of 50 cells/cm^2^. The treated and untreated cells were maintained in fresh medium for 14–21 days. Then, the cells were fixed in 100% methanol, and stained with 0.5% crystal violet in 20% methanol. Therefore, the plates were air dried. The colonies were photographed using a digital camera and counted by using image master 2D software. Colonies containing more than 50 cells were counted, that corresponds about to a volume of 19,000 pixel. Results are expressed as percentage relative to NT sample.

### Statistics

Data were expressed as mean ± SD and analysed by ANOVA or by Paired *t-*test. A *p* value of less than 0.05 was considered as statistically significant.

## Results

### Composition of FC from Volcano Island

The composition of FC collected from the crater of Volcano Island was similar to that previously reported [15, 22] and contained prevalently water vapor, followed by carbon dioxide and sulfur species. Among acid volcanic gases, sulfur species and in particular H_2_S and SO_2_ are present (Table 1).

**Table 1:** Analysis of FC from fumarole F5HT of Vulcano Island (data in vol %)

Date	12–05–2016	T °C	352
H_2_O	93.50	O_2_	6.14E–06
CO_2_	6.24	N_2_	0.03
HF	0.01	CO	2.25E–06
HCl	0.02	CH_4_	4.3E–05
S tot*	0.22	Ne	1.6E–06
H_2_S	8.16E–03	Ar	1.07E–03
He	3.8E–05		

*Total amount of sulfur due to the following components: S + H_2_S + SO_2_

### Effects of FC on IL-33 expression in 16HBE and A549 cells

**Fig. 1:**
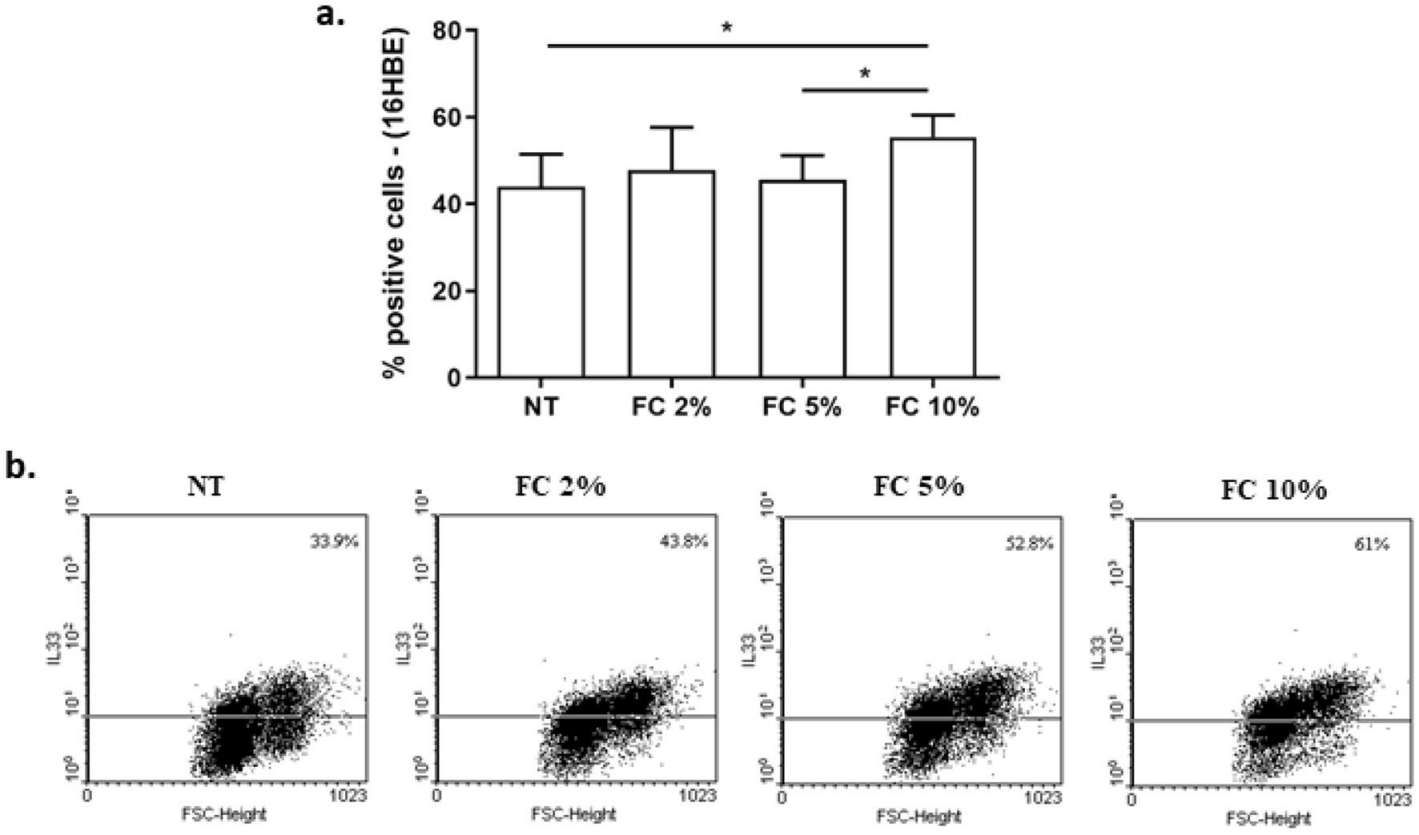
Effects of FC on intracellular IL-33 in 16HBE. 16HBE were cultured and stimulated with different concentrations of FC (2–5–10%) and then assessed for intracellular IL-33 protein by Flow cytometry. **a** Data are expressed as % of positive cells (mean ± SD) (*n* = 4). *p < 0.05 (paired *t* test). **b** Representative dot plots were shown

**Fig. 2:**
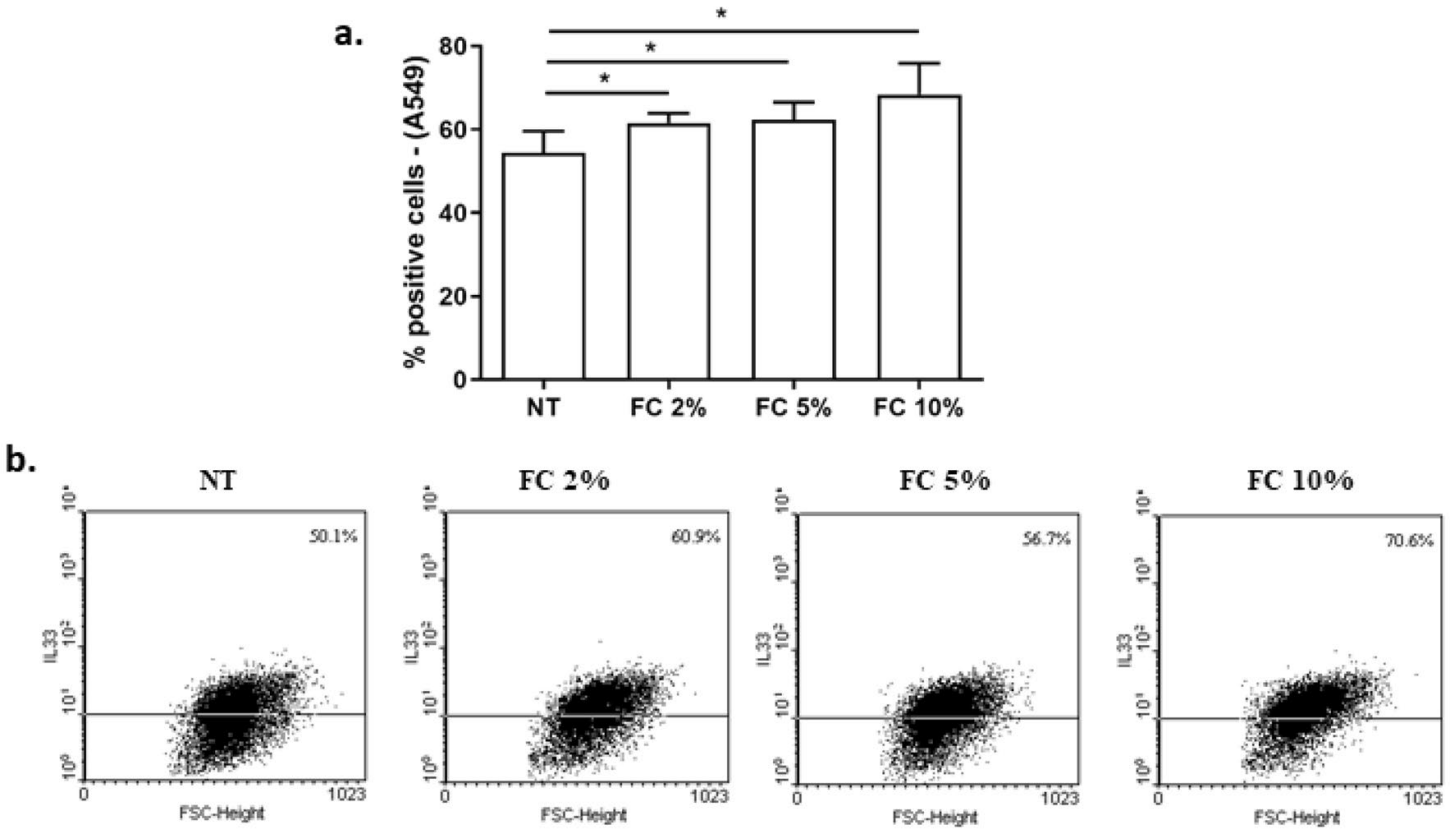
Effects of FC on intracellular IL-33 in A549. A549 were cultured and stimulated with different concentrations of FC (2–5–10%) and then assessed for intracellular IL-33 protein by Flow cytometry. **a** Data are expressed as % of positive cells (mean ± SD) (*n* = 4). *p < 0.05 (paired *t* test). **b** Representative dot plots were shown

Airway epithelial cells express high intracellular levels of IL-33 and release relevant amount of IL-33 upon cell necrosis [23]. Initially, we tested the effect of FC at different concentrations (2%, 5% and 10%) on intracellular expression of IL-33 in 16HBE and A549 cells. As shown in Figs. 1a–b and 2a–b, FC increased intracellular IL-33 expression in both cell lines in a dose-dependent manner and FC at 10% exerted the highest effect on intracellular increase of IL-33.

**Fig. 3:**
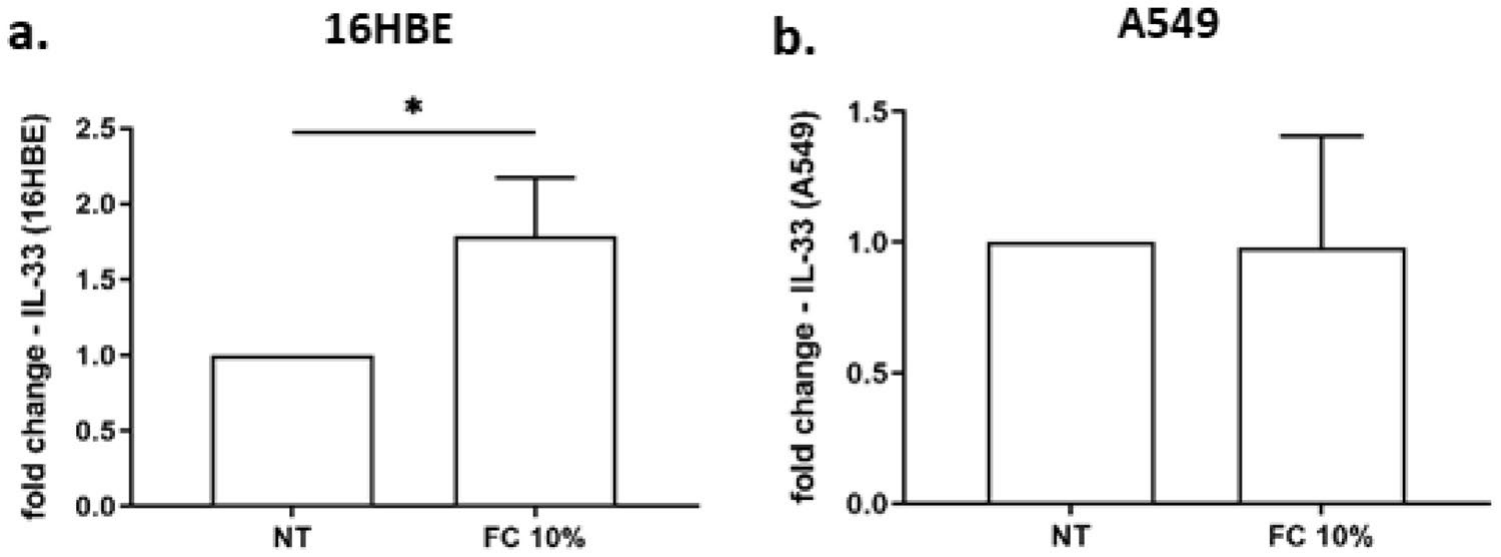
Effects of FC on IL-33 gene expression of 16HBE and A549. 16HBE (**a**) and A549 (**b**) were cultured and stimulated with FC 10% and then assessed for IL-33 gene expression by real-time PCR. GAPDH gene expression was used as endogenous control for normalization. Relative quantitation of mRNA was carried out with comparative CT method. Results are reported as relative units and normalized to no treated (NT) control. *p < 0.05 (paired *t* test)

To explore whether the increase of IL-33 protein upon FC exposure was due to increased gene expression, the IL-33 gene expression was also evaluated. FC was able to significantly induce IL-33 gene expression in 16HBE but not in A549 (Fig. 3a–b), suggesting a different regulation of IL-33 protein expression in the two cell lines.

**Fig. 4:**
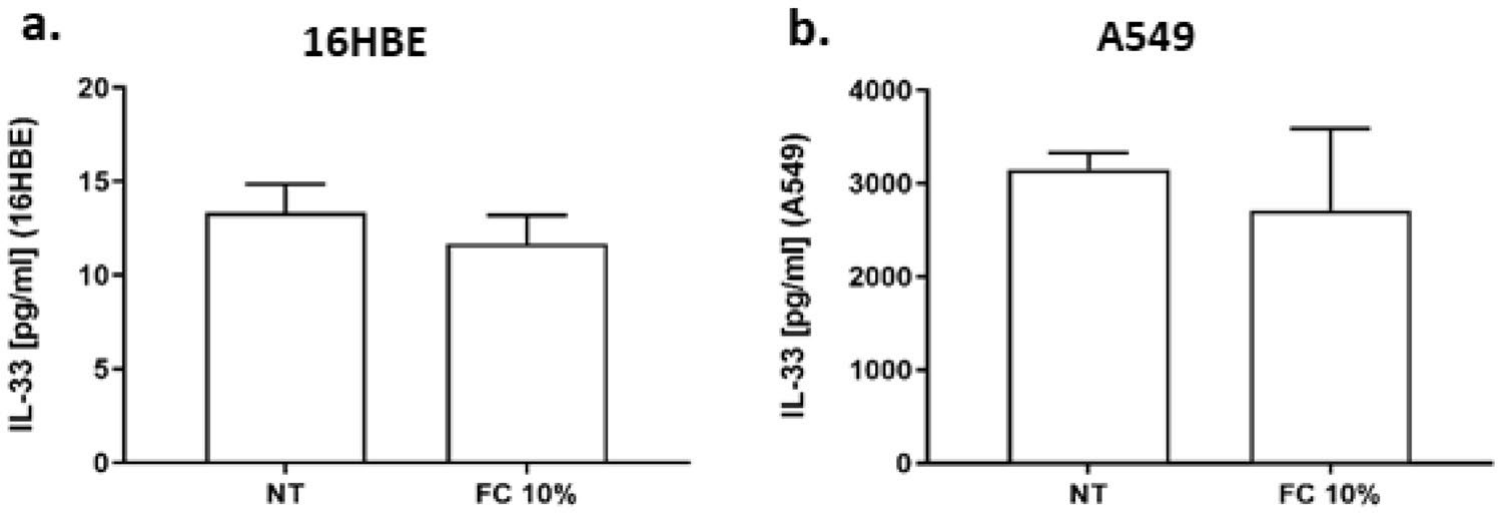
Effects of FC IL-33 release in 16HBE and A549. 16HBE (**a**) and A549 (**b**) were cultured and stimulated with FC 10% and then assessed for IL-33 release by ELISA. Data are expressed as IL-33 concentration in pg/ml (mean ± SD) (*n* = 4). *p < 0.05 (paired *t* test)

Since IL-33 is an allarmin that when released by necrotic cells promotes Th2 responses [23], the effect of FC on IL-33 release was assessed. Data shown in Fig. 4a–b for both cell lines, demonstrated that FC did not modify the release of IL-33.

### Effects of FC on IL-8 gene expression and release in 16HBE and A549 cells

**Fig. 5:**
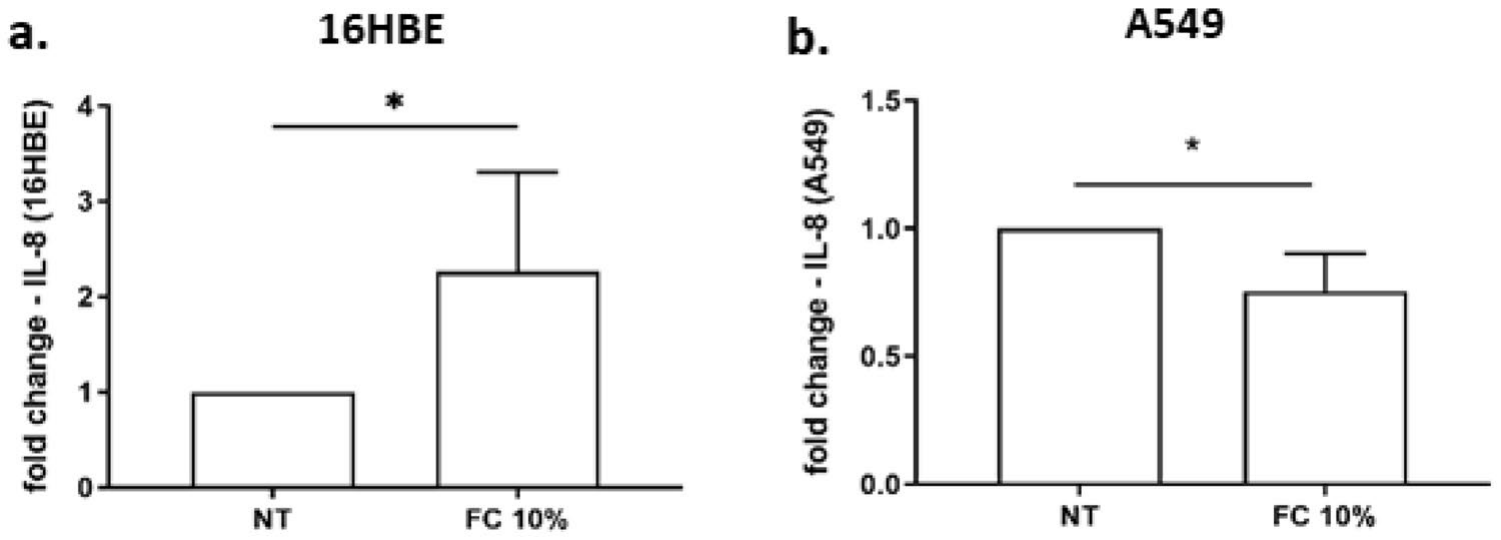
Effects of FC on IL-8 gene expression of 16HBE and A549. 16HBE (**a**) and A549 (**b**) were cultured and stimulated with FC 10% and then assessed for IL-8 gene expression by real-time PCR. GAPDH gene expression was used as endogenous control for normalization. Relative quantitation of mRNA was carried out with comparative CT method. Results are reported as relative units and normalized to no treated control (NT). *p < 0.05 (paired *t* test)

**Fig. 6:**
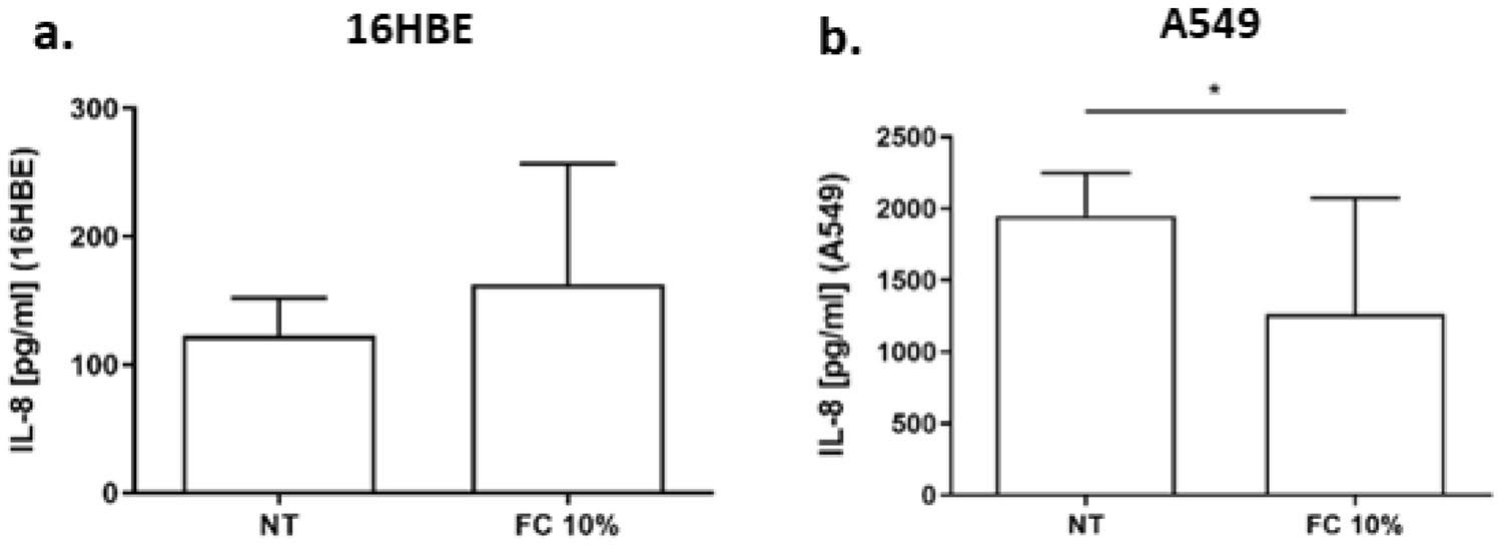
Effects of FC IL-8 release in 16HBE and A549. 16HBE (**a**) and A549 (**b**) were cultured and stimulated with FC 10% and then assessed for IL-33 release by ELISA. Data are expressed as IL-8 concentration in pg/ml (mean ± SD) (*n* = 4). *p < 0.05 vs NT (paired *t* test)

IL-8 is a chemokine that exerts a relevant pro-inflammatory role in recruiting neutrophil within the airways [24]. Thus, the effect of FC on IL-8 was assessed. The results show that FC exposure significantly increased IL-8 gene expression in 16HBE (Fig. 5a), but FC significantly reduced IL-8 gene expression in A549 cells (Fig. 5b). Accordingly, FC was able to significantly reduce IL-8 release in A549 (Fig. 6b) but not in 16HBE (Fig. 6a).

### Effects of FC and CSE, alone and combined, on cell viability/metabolism in 16HBE and A549 cells

**Fig. 7:**
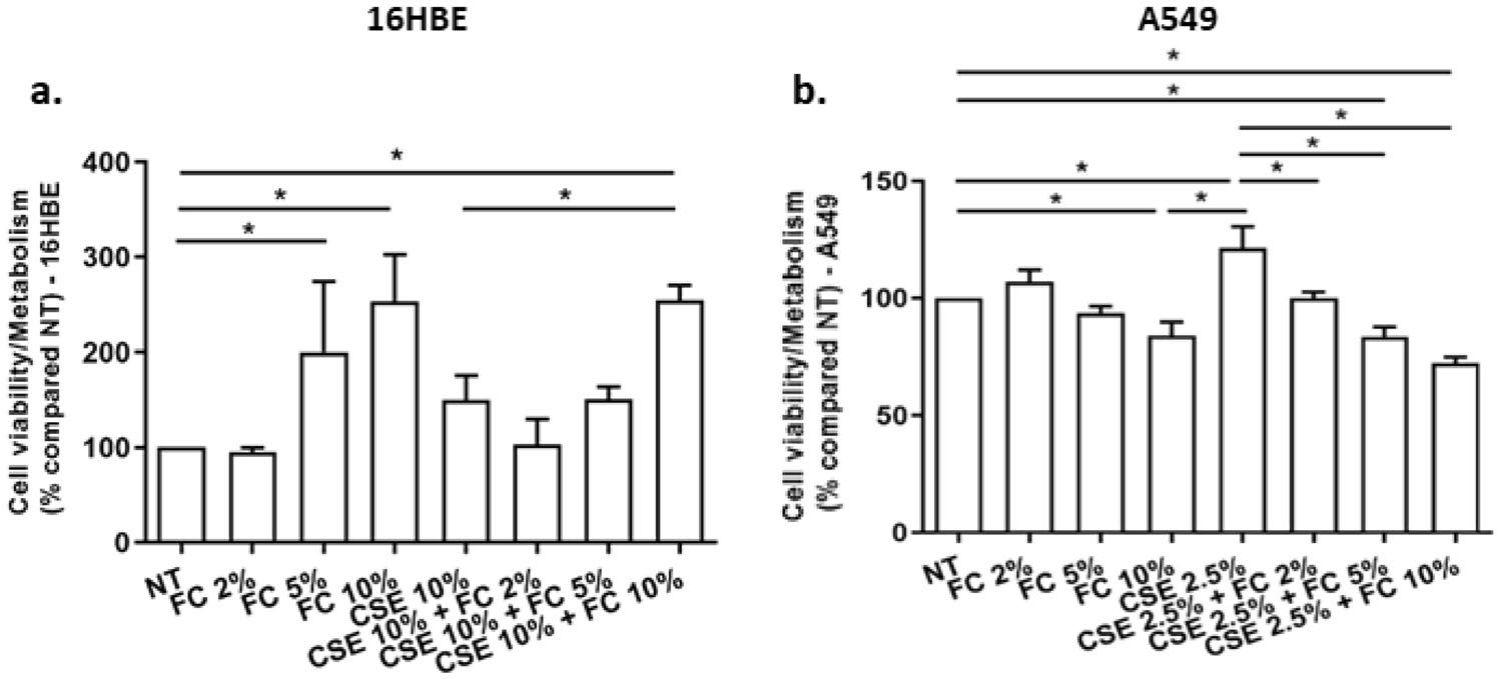
Effects of FC and CSE on cell viability/metabolism in 16HBE and A549. 16HBE (**a**) and A549 (**b**) were cultured and stimulated with FC (2, 5 and 10%) and with CSE (10% for 16HBE and 2.5% for A549) and then assessed for cell viability/metabolism by MTS assay. Results are expressed as percentage relative to no treated (NT) sample. Data represent mean ± S.D. (*n* = 3). *p < 0.05 (ANOVA)

Cigarette smoke is the main risk factor for chronic inflammatory airway diseases [6]. We next wondered whether FC alone or combined with CSE altered cell viability/metabolism of 16HBE and of A549 airway epithelial cell lines by MTS. As shown in Fig. 7a, FC 10% significantly increased cell viability/metabolism, while CSE 10% did not significantly alter cell viability/metabolism in 16HBE. A significant increase in cell viability/metabolism is observed in FC 10% + CSE 10% 16HBE-treated cells in comparison to untreated cells or to CSE-treated cells. Differently, FC 10% reduced cell viability/metabolism in A549 (Fig. 7b). CSE 2.5% increased cell viability/metabolism and the addition of FC (2, 5 and 10%) significantly reduced cell viability/metabolism in cells treated with CSE (Fig. 7b) in A549 cells.

### Effects of FC and CSE, alone and combined, on cell apoptosis/necrosis in 16HBE and A549 cells

**Fig. 8:**
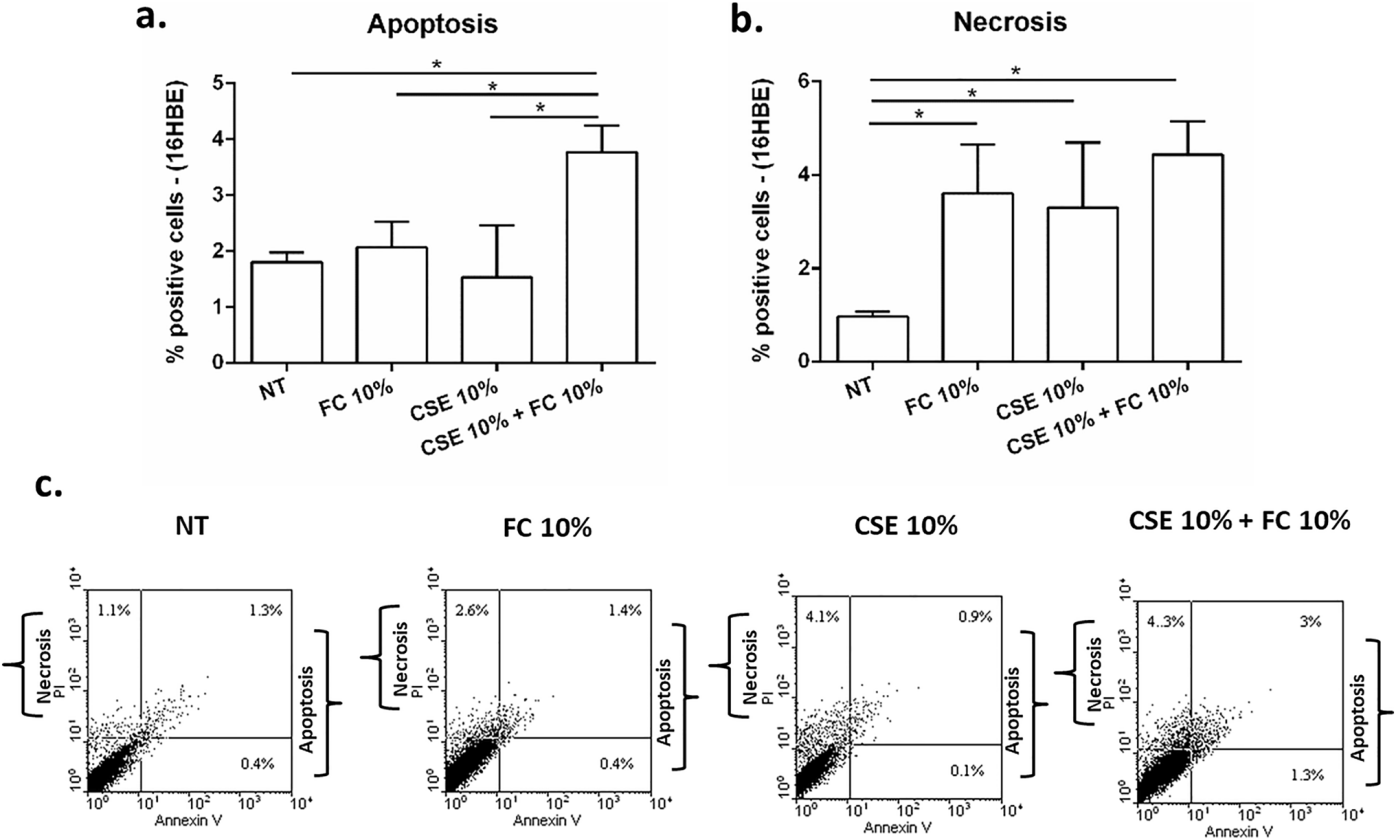
Effects of FC and CSE on cell apoptosis and cell necrosis in 16HBE. 16HBE were cultured and stimulated with FC 10% and with CSE 10% and then assessed for cell apoptosis (**a**) and cell necrosis (b) by Annexin V/PI method. **a**–**b** Data represent mean ± S.D. (*n* = 3). *p < 0.05 (ANOVA). **c** Representative dot plots were shown

**Fig. 9:**
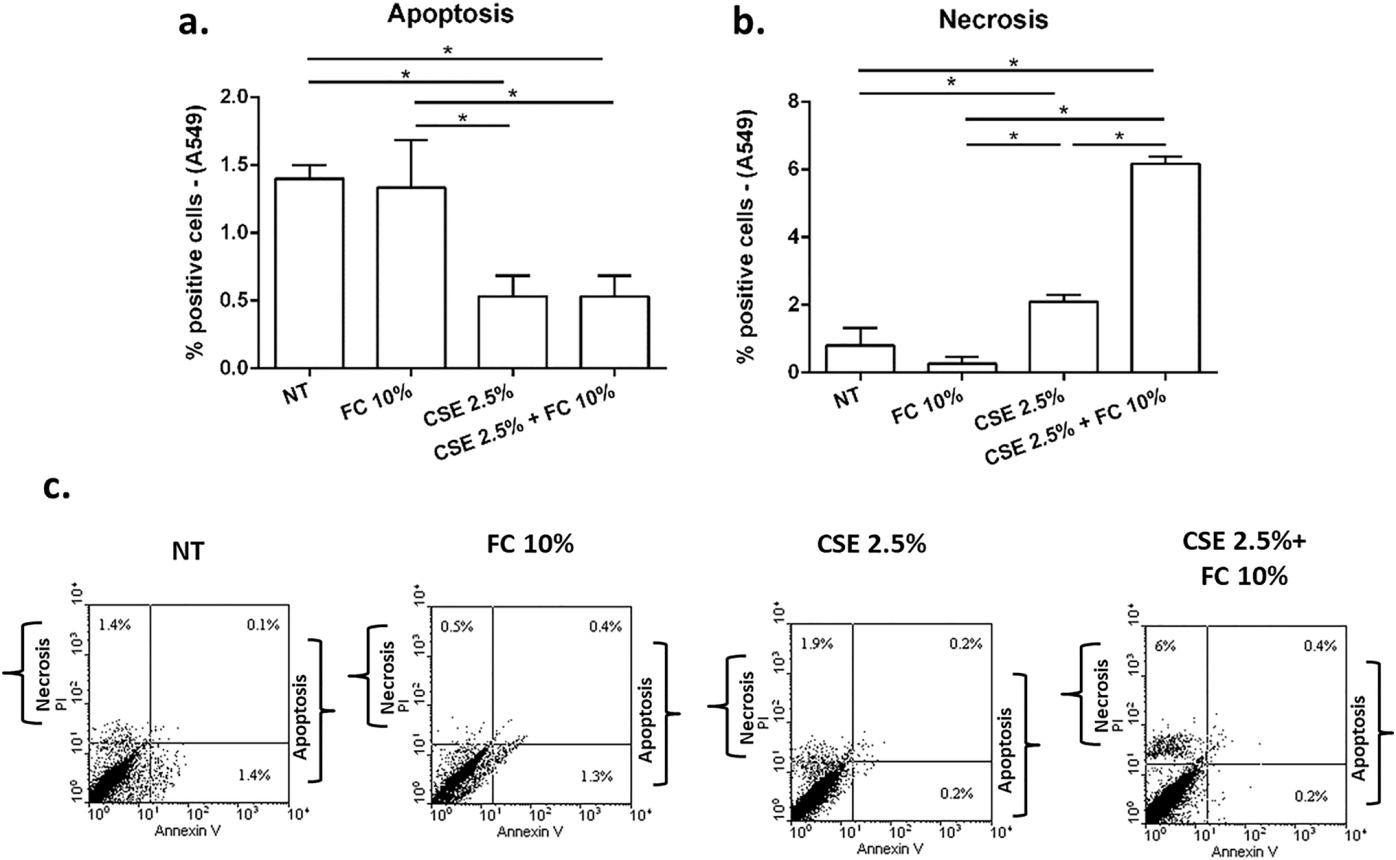
Effects of FC and CSE on cell apoptosis and cell necrosis of A549. A549 were cultured and stimulated with FC 10% and with CSE 2.5% and then assessed for cell apoptosis (**a**) and cell necrosis (**b**) by Annexin V/PI method. **a**–**b** Data represent mean ± S.D. (*n* = 3). *p < 0.05 (ANOVA). **c** Representative dot plots were shown

In order to understand whether the alterations of cell viability/metabolism observed in 16HBE and in A549 exposed to FC and CSE was due to cell death, we further assessed the effect of these stimuli on cell apoptosis/necrosis by Annexin V/PI method. FC 10% did not significantly modify cell apoptosis in both cell lines (16HBE—Fig. 8a–c; A549—Fig. 9a–c). While FC 10% induced a modest but significant increase in cell necrosis in 16HBE (Fig. 8b–c) and slightly but not significantly decreased cell necrosis in A549 (Fig. 9b–c). CSE 10% did not affect cell apoptosis but significantly induced necrosis in 16HBE. The treatment with the combination of FC 10% and CSE 10% significantly increased cell apoptosis and necrosis in 16HBE (Fig. 8a, b and c). CSE 2.5% alone and in combination with FC 10% significantly reduced cell apoptosis in A549 (Fig. 9a–c). CSE 2.5% alone slightly increased cell necrosis in A549 but the combination with FC 10% induced a more significant increase in comparison to NT and CSE treated A549 (Fig. 9b–c).

### Effects of FC and CSE, alone and combined, on mitochondrial superoxide production in 16HBE and A549 cells

**Fig. 10:**
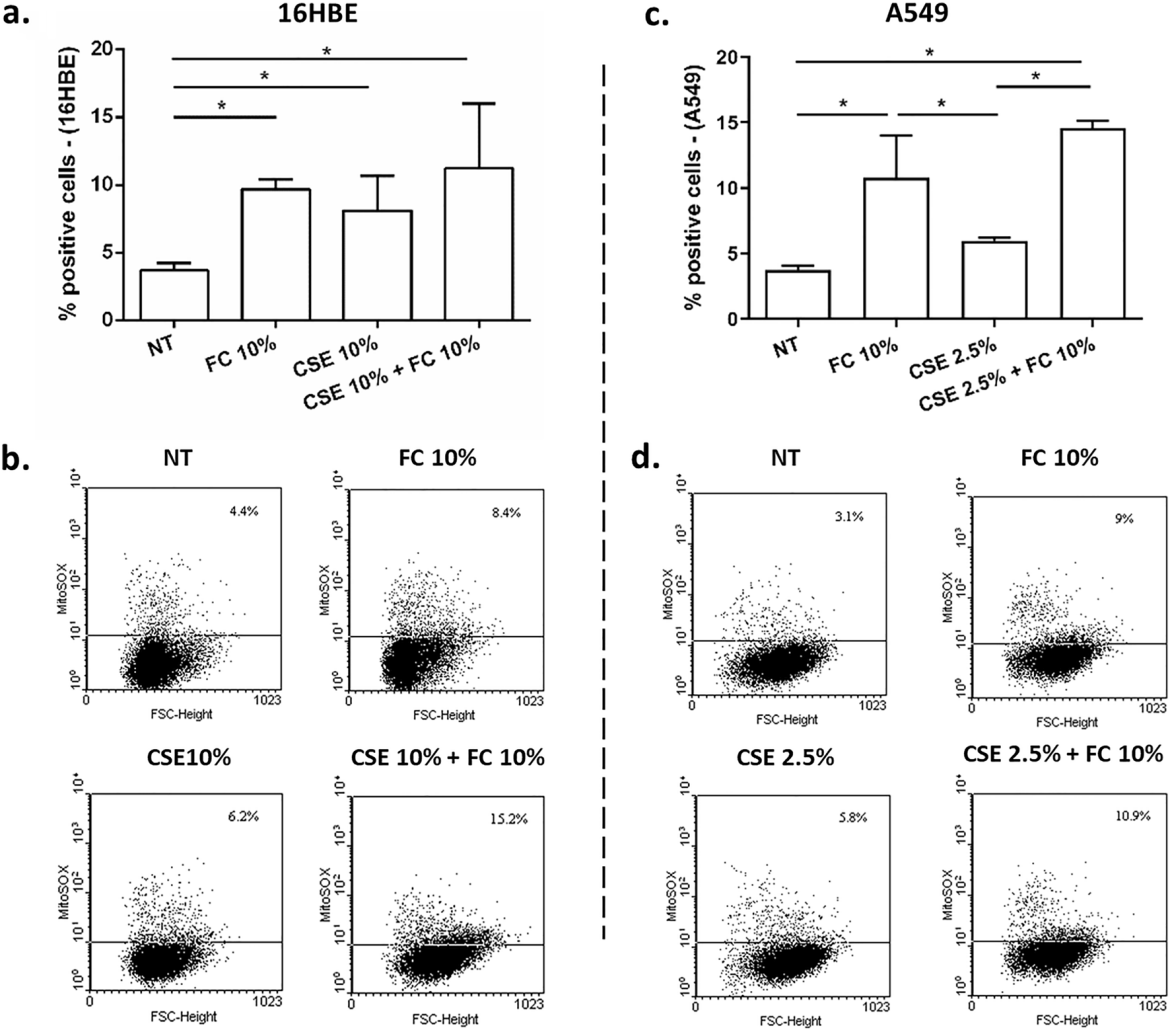
Effects of FC and CSE on mitochondrial superoxide production in 16HBE and A549. 16HBE (**a**–**b**) and A549 (**c**–**d**) were cultured and stimulated with FC 10% and with CSE (10% for 16HBE and 2.5% for A549) and then assessed for mitochondrial superoxide production by Flow Cytometry. **a**–**c** Data are expressed as % of positive cells (mean ± SD) (*n* = 4). *p < 0.05 (ANOVA). **b**–**d** Representative dot plots were shown

Since IL-33 intracellularly is increased in FC-exposed cells as well as in CSE-exposed cells [7] and since IL-33 increases in vitro mitochondrial membrane potential [25], we assessed the effects of FC, CSE and CSE + FC on the mitochondrial superoxide production. As shown in Fig. 10, FC 10%, CSE and CSE + FC significantly increased the mitochondrial superoxide production in both 16HBE (Fig. 10a–b) and A549 (Fig. 10c–d) suggesting that the different effect of FC in cell viability/metabolism of 16HBE and A549 was not due to a different effect in mitochondrial superoxide production.

### Effects of FC and CSE, alone and combined, on long-term proliferation in 16HBE and A549 cells

**Fig. 11:**
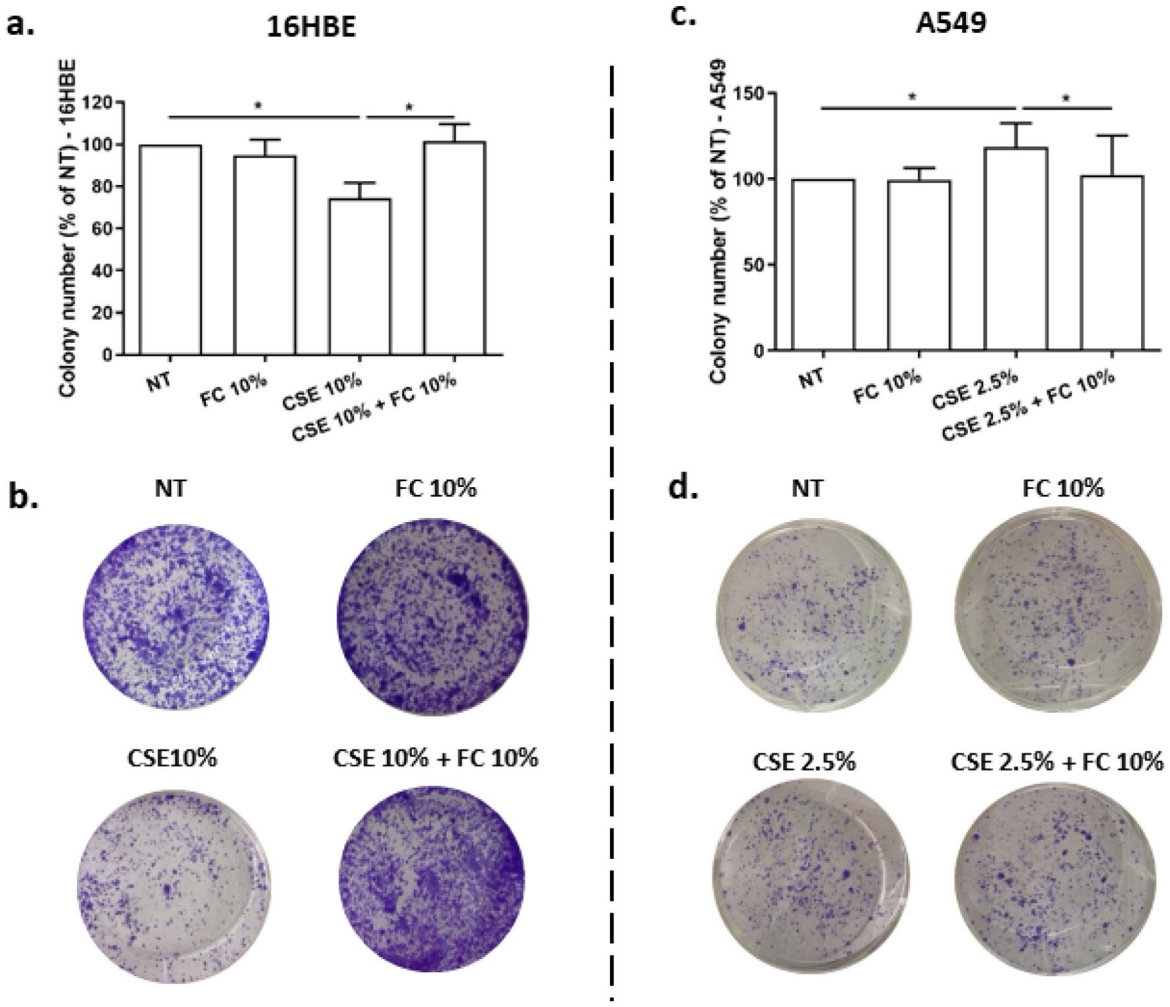
Effects of FC and CSE on long-term cell proliferation of 16HBE and A549. 16HBE (**a**–**b**) and A549 (**c**–**d**) were cultured and stimulated with FC 10% and with CSE (10% for 16HBE and 2.5% for A549) and then assessed for cell proliferation by clonogenic assay. **a**–**c** Data (mean ± SD) are expressed as percentage relative to untreated cells (NT) (100%) (*n* = 3). *p < 0.05 (ANOVA). **b**–**d** Representative images were shown

The long-term effects of FC, CSE and FC + CSE exposure were evaluated in 16HBE and A549 assessing cell proliferation (clonogenic activity). FC 10% did not modify cell proliferation in both cell lines (Fig. 11a–d). CSE significantly reduced cell proliferation in 16HBE while increased it in A549 (Fig. 11a–d). The treatment with FC and CSE in combination restored the normal cell proliferation in 16HBE (Fig. 11a–b) and in A549 (Fig. 11c–d).

## Discussion

Exposure to abiotic insults present in the environacument including volcanic emissions could be detrimental to the structural integrity and functioning of the respiratory system and can threaten the health of inhabitants in many ways. It is well known that air pollution due to volcanic activity including volcanic ash, exerts a negative impact on immunological defenses, increases the occurrence of biological redox reactions and induces symptoms of cough, phlegm, and wheeze and disease exacerbations in people who suffer from chronic airway diseases [26]. A previous paper on mice captured in villages with active volcanism and compared to those captured in a village without any type of volcanic activity, demonstrates that mice exposed to volcanogenic activity present bronchioles with increased epithelial thickness, increased smooth muscle layer, increased submucosa thickness and increased peribronchiolar inflammation [27]. Hydrothermal emissions are associated with chronic bronchitis [28], and increased prevalence of respiratory restrictions and obstructions [29]. However, the effects of exposure to hydrothermal emissions such as volcanic fumaroles alone or combined with other toxic inhalants on human health are largely unknown.

Among toxic inhalants, Cigarette Smoke (CS) represents the major risk factor for the main lung diseases including COPD and lung cancer. In airway cellular models, CSE promote alterations of the injury/repair processes [30], senescence processes [31], inflammatory responses [32] and a pro-tumorigenesis program [9].

The present study was designed to assess, in vitro, the effects of condensate of volcanic fumaroles (FC), collected from La Fossa crater of Vulcano Island, alone or combined with CSE on airway epithelial cells originating from proximal (16HBE) and distal (A549) airways. The fumaroles are mainly composed of water vapor and also of carbon dioxide, hydrogen sulfide and sulfur dioxide and other volcanic gases (hydrochloric acid, carbon monoxide and hydrofluoric acid). While hydrogen sulfide exerts some beneficial effects, sulfur dioxide can cause breathing problems in both healthy people and people with asthma and other respiratory problems.

Hydrogen sulfide (H_2_S) exerts a novel antiviral and anti-inflammatory activity in mice infected with RSV [28, 33] and owns antioxidant and anti-inflammatory skills, involving in the pathophysiological process of COPD [34]. H_2_S mitigates pro-remodeling effects of CS upregulating SIRT1 expression and inhibiting the activation of TGF-β1/Smad3 signaling in mice animal models [35]. Conversely, sulfur dioxide (SO_2_) is a pollutant present in automobile fumes and inhaled SO_2_ easily forms its soluble derivatives (bisulfite and sulfite) which are toxic to the respiratory system and may play a role in the exacerbation of airway disease symptoms. Previous evidences have demonstrated that SO_2_ derivatives can induce mucus over-production and inflammation responses in human bronchial epithelial cells by increasing MUC5AC and IL-13 gene and protein expression [36]. Other potential toxic effects could be related to increased levels of CO_2_. Variations in pH of airway epithelia may occur in vivo in response to shifting luminal CO_2_ tension. La Fossa fumaroles are composed of gases rich in CO_2_ [37] whose concentration varies from 3 to 30%. In this regard a previous paper has demonstrated that CO_2_ (10 and 20%) promotes the release of serotonin via carbonic anhydrase mRNA and protein induction [38]. Furthermore, it has been demonstrated that reduced extracellular pH reduces ciliary beating frequency [39] and similar effects are observed when cell cultures were exposed to SO_2_ [40]. These potential negative effects on ciliary function may expose to a further increased risk of airway injury: ciliated cells move the layer of mucus containing the airborne insults towards the epiglottis, thus away from airspaces. Speculatively, fumaroles, based on their composition, may potentially exert both detrimental and beneficial effects on airway epithelial cells of people who live in a volcanic area.

The airway tract mucosa plays an important role in protecting the lungs from environmental insults and maintaining homeostasis [8]. The airway can be divided into two zones: the conducting zone (proximal airways) and the respiratory zone (distal airways and parenchyma). The conducting zone is deputed to modify inhaled air by moistening, warming, and cleaning before it reaches the lower respiratory zone, where gas exchange occurs [8].

Herein, we designed an experimental model in which airway cells were exposed to different concentrations of FC (2, 5 and 10%) to better reproduce the airways exposure to different levels of gases contained in the fumaroles. This is because people who live near the volcano or who are downwind may be exposed to higher fumarole concentrations which can affect health differently if the exposure is less intense. Furthermore, in our models, the used cell lines were also exposed to CSE as a reference toxic stimulus for the airway models and to mimic some molecular events and cellular responses in smokers’ airways.

We explored the impact of FC alone on IL-33 and IL-8 in both cell lines. IL-33 is an alarmin, abundant in the nuclei of tissue-derived cells, including epithelial cells of the airways and released upon cell necrosis or tissue injury [7]. The effects of extracellular IL-33 are mediated by the activation of Myd88-dependent signaling pathways upon interaction with membrane the ST2 (IL-1RL1) receptor [23]. In the present study, we observed an increase in IL-33 gene expression in 16HBE but not in A549. FC increased intracellular IL-33 in both cell lines thus suggesting a role of IL-33 as transcription factor as previously reported [7]. In terms of extracellular IL-33, no significant variations of IL-33 were found after 24 h of FC exposure in both cell lines. This phenomenon could be due to the finding that although IL-33 is rapidly released upon cell damage and tissue injury, the protein is no longer detectable in extracellular fluids after a few hours [41]. In addition, FC slightly induced cell necrosis, a mechanism with a crucial role in the extracellular release of IL-33.

IL-8 is a chemokine with a crucial role in the recruitment of neutrophils and in the injury of the airways [24] and is also a cytokine promoting pro-cancerous events [42]. Here, no relevant effect was observed for the modulation of IL-8 protein release in 16HBE. In A549, it was demonstrated that IL-8 gene expression and IL-8 release were both reduced by FC. Differently, it has been previously reported [9] that CSE in A549 induces both IL-8 gene expression and release, suggesting a different impact of FC and CSE on IL-8. Furthermore, here we evaluated the combined effects of FC and CSE on cell viability/metabolism, mitochondrial superoxide production, cell death and cell proliferation. We demonstrate that FC 10% significantly increased cell viability/metabolism and this effect persisted also in CSE exposed 16HBE cells. Differently in the A549, FC decreased cell viability/metabolism and this effect is confirmed also when the cells were exposed to the combination of FC and CSE. Of note, for this evaluation, we used the MTS assay whose reduction reflects vital cellular metabolism and relies on mitochondrial NAD(P)H-dependent oxidoreductase enzymes to convert tetrazole to formazan [43]. Therefore, the number of viable cells and their metabolic activity can be evaluated with this method. Furthermore, superoxide production can be determined by levels of the mitochondrial NADH/NAD + ratio [44]. In the present study, FC is associated with increased mitochondrial superoxide production in both cell lines. Furthermore, we confirmed that CSE induced mitochondrial superoxide production in both cell types [19, 45] and the combination of FC with CSE maintains the increased mitochondrial superoxide production. Therefore, the positive effect on the control of cell viability/metabolism is associated with increased mitochondrial oxidative stress in 16HBE but not in A549. The potential toxic effects of FC alone or combined with CSE were also explored by studying cell death. Cell apoptosis and necrosis were assessed by annexin V/PI method. Annexin V, a 36-kDa calcium-binding protein, binds to phosphatidylserine that is exposed on the outside of apoptotic cells. Apoptotic cells can be distinguished from necrotic cells by co-staining with propidium iodide (PI), because PI enters necrotic cells but is excluded from apoptotic cells [46]. Our results showed that F.C did not induce cell apoptosis in both the cell types while it increased cell necrosis in 16HBE and reduced cell necrosis in A549. CSE alone reduced cell apoptosis while it slightly increased cell necrosis in A549. The combination of FC and CSE induced cell necrosis and apoptosis in 16HBE and cell necrosis in A549. Since cell viability/metabolism, apoptosis/necrosis and mitochondrial superoxide production were all effects assessed few hours (24 h) after FC and CSE exposure, to further explore the effects of FC and CSE on airway epithelial cell homeostasis, the long-term (15–21 days) proliferation by clonogenic assay was also tested. Both proximal and distal airway epithelial cells cultured with FC maintain their proliferative potential further upon acute exposure. However, CSE exposure reduced the proliferative potential of 16HBE and the co-exposure to FC restored this capability of the cells. Otherwise, CSE increased proliferative potential and FC counteracted this CSE mediated effect in A549.

## Conclusions

Data provided by the present study support the notion that FC contained gases with opposite effects that could differently act on proximal and distal airway epithelial cells originating from different geographical districts of the airways. Our results showed that, on one hand, the exposure to fumaroles modulates the pro-inflammatory profile of both cell lines increasing IL-33 intracellular expression in both cell lines but reducing IL-8 (gene and protein release) only in A549. FC exert a reparative effect upon CSE mediated injury in proximal epithelial cells. Further studies are warranted to assess whether these molecular events described in this in vitro setting of acute exposure were also observed, in vivo, in a cohort of habitants of Vulcano Island who are chronically exposed to fumaroles.

## Supplementary Information

Below is the link to the electronic supplementary material.

Supplementary file1 (DOCX 15 KB)

## Data Availability

The data used to support the findings of this study are included within the article.

## Abbreviations

FC: Fumarole condensate
H_2_O: Water
CO_2_: Carbon dioxide
HF: Hydrofluoric acid
HCl: Hydrochloric acid
S tot: Total amount of sulfur due to the following components: S + H_2_S + SO_2_
SO_2_: Sulfur dioxide
H_2_: Hydrogen
He: Helium
O_2_: Oxygen
N_2_: Nitrogen
CO: Carbon monoxide
CH_4_: Methane
Ne: Neon
Ar: Argon
CSE: Cigarette smoke extract
