# Supplementary material for: Effects of condensates from volcanic fumaroles and cigarette smoke extracts on airway epithelial cells
Source: Hum Cell. 2023 Jun 12;36(5):1689–702. doi: 10.1007/s13577-023-00927-1 (PMC10390407; doi:10.1007/s13577-023-00927-1)
Supplement: Supplementary file 1 — Supplementary file1 (DOCX 15 KB) [file 13577_2023_927_MOESM1_ESM.docx]

**Supplementary Materials (S1)**

**Method for collecting Acid Condensate of fumarole**

Immediately before taking the gas, the temperature of the sampling site is measured by a thermocouple, which is inserted into the ground for a few decimeters. A metal foil, composed by an external part and an internal core, is inserted into the ground along its entire length. The external part consisted of a metal tube with a diameter of 3 cm and a length of 60 cm, while the internal part consisted of a pointed rod with a diameter of approximately 1 cm and of the same length as the foil. Upon the metal foil is inserted into the ground, the core is extracted, then the dewar tube is inserted inside the foil. The dewar tube is a pyrex glass tube with an external chamber within which the vacuum has been practiced and an internal part through which the gas can flow. If the gaseous flow is not coming out, it is possible to facilitate the exit of the gas by aspirating through a syringe. With the metal tweezers or a silicone connector tube, the dewar is connected to an ampoule. It is a cylindrical container in pyrex glass equipped with two taps and containing 50 ml of an alkaline solution (sodium hydroxide: NaOH 4 molar); the ampoule is thus partially filled; subsequently, a vacuum is created within it which will have the very important function of drawing the fumarolic gas into the ampoule. The ampoule is weighed before and after sampling and the difference obtained is equal to the weight of the sample collected.

The dewar tube has the function of conveying the gas into the interrupter and its vacuum chamber to prevent the gas from being excessively cooled during this passage and therefore condensing before reaching the interrupter (it should be remembered that 90% of the fumarolic gas is water vapor). The connection between the ampoule and the dewar must be such as to prevent any intrusion of air into the system and, for this reason, care must be taken to fill the tap from which the gas enters with millipore water. Only when you are sure of the correct connection between the ampoule and the dewar can you open the tap and allow the gas to enter. The gas that enters the ampoule bubbles into the solution: acid gases (HCl, HF, H2S, SO2, CO2) undergo salification reactions remaining trapped in the basic solution; the non-condensable gases (He, H2, O2, N2, CO, CH4) instead pass through the solution and accumulate in the remaining part of the bulb going to slowly occupy the vacuum space. When the gas pressure within the ampoule equals that of the incoming gas flow, the bubbling ends and sampling must be stopped by closing the tap. At this point the sampling can be considered concluded.
